# The association between prediabetes and obstructive sleep apnea syndrome: a meta-analysis

**DOI:** 10.3389/fendo.2026.1798827

**Published:** 2026-07-30

**Authors:** Lili Miao, Quanming An, Shaojin Wang, Rui Zhang

**Affiliations:** 1 Department of Pulmonary and Critical Care Medicine, General Hospital of Ningxia Medical University, Yinchuan, Ningxia, China; 2 Department of Gastrointestinal Surgery, General Hospital of Ningxia Medical University, Yinchuan, Ningxia, China

**Keywords:** hyperglycemia, intermittent hypoxia, meta-analysis, obstructive sleep apnea syndrome, prediabetes

## Abstract

**Background:**

Obstructive sleep apnea syndrome (OSAS) has been increasingly linked to metabolic dysfunction. However, the association between prediabetes and OSAS remains incompletely defined. This meta-analysis aimed to systematically evaluate the relationship between prediabetes and the prevalence of OSAS in adults.

**Methods:**

PubMed, Embase, and Web of Science were systematically searched to identify observational studies evaluating the association between prediabetes and objectively diagnosed OSAS. Odds ratios (ORs) with 95% confidence intervals (CIs) were pooled using random-effects models accounting for the influence of possible heterogeneity.

**Results:**

Eight cross-sectional studies comprising 18,040 participants were included, of whom 6,325 (35.1%) had prediabetes. Overall, individuals with prediabetes exhibited a significantly higher prevalence of OSAS compared with normoglycemic controls (OR = 2.78, 95% CI 1.99–3.87; p < 0.001), with substantial heterogeneity (I² = 83%). Sensitivity analyses confirmed the robustness of the findings (OR range: 2.47–3.08). Consistent associations were observed across Asian and Western populations, community-derived and high-risk populations, and across different diagnostic methods for OSAS. A stronger association was noted among studies with younger populations (mean age < 55 years vs. ≥ 55 years: OR 3.44 vs. 2.06; *p* for subgroup difference = 0.04) and when prediabetes was defined using composite diagnostic criteria rather than single criteria (OR 3.40 vs. 1.92; *p* = 0.01).

**Conclusions:**

Prediabetes is significantly associated with a higher prevalence of OSAS in adults. These findings highlight the frequent coexistence of early glucose dysregulation and sleep-disordered breathing and support consideration of OSAS screening in individuals with prediabetes.

**Systematic review registration:**

, identifier CRD420261296015.

## Introduction

Obstructive sleep apnea syndrome (OSAS) is a common sleep-related breathing disorder characterized by recurrent upper airway collapse during sleep (1, 2). These events lead to intermittent hypoxia, sleep fragmentation, and fluctuations in intrathoracic pressure (1, 2). Epidemiological studies estimate that OSAS affects approximately 10–30% of adults worldwide, with a substantial proportion remaining undiagnosed (2). Beyond its well-recognized impact on sleep quality and daytime functioning, OSAS has been increasingly identified as an important contributor to cardiometabolic morbidity (3). Accumulating evidence links OSAS to hypertension, cardiovascular disease, and metabolic disorders, independent of traditional risk factors (4). Mechanistically, intermittent hypoxia and sleep disruption induce sympathetic activation, systemic inflammation, oxidative stress, and hormonal dysregulation, all of which play critical roles in the development of insulin resistance and impaired glucose homeostasis (5). Consequently, OSAS is now considered not merely a respiratory disorder but a systemic disease with profound metabolic consequences (5, 6).

Prediabetes represents an intermediate metabolic state between normoglycemia and overt diabetes mellitus and is commonly defined by impaired fasting glucose (IFG), impaired glucose tolerance (IGT), or mildly elevated glycated hemoglobin (HbA1c) levels (7). Globally, the prevalence of prediabetes has risen rapidly, affecting more than one-third of adults in many populations (7, 8). Importantly, prediabetes is not a benign condition; individuals with prediabetes face a substantially increased risk of progression to type 2 diabetes (T2D) (9), as well as early vascular (10) and metabolic complications (11). Without timely intervention, an estimated 5–10% of individuals with prediabetes convert to diabetes annually (12). Identifying modifiable factors that contribute to metabolic deterioration during this early stage is therefore of major clinical importance. Emerging studies suggest that OSAS may adversely influence glycemic metabolism even before diabetes develops, through pathways involving insulin resistance, β-cell dysfunction, and altered glucose regulation (13).

In recent years, increasing attention has been directed toward the relationship between prediabetes and OSAS. Several observational studies have reported a higher prevalence of OSAS among individuals with prediabetes compared with normoglycemic populations, although the magnitude of this association has varied across studies (14–21). Importantly, interventional evidence indicates that continuous positive airway pressure (CPAP) therapy may improve insulin sensitivity and glycemic parameters in patients with OSAS (22, 23), supporting a potential role of sleep-disordered breathing in metabolic dysregulation. If prediabetes is closely associated with OSAS, early recognition of coexisting sleep apnea may represent a valuable opportunity to prevent further deterioration of glucose metabolism and delay progression to overt diabetes. However, existing studies are heterogeneous in design, population characteristics, and diagnostic criteria, and no comprehensive quantitative synthesis has been performed to clarify this association (14–21). Therefore, the present meta-analysis systematically evaluates the association between prediabetes and OSAS in adult populations and aims to provide evidence supporting early identification and metabolic risk stratification in individuals with early glucose dysregulation.

## Methods

This meta-analysis was conducted and reported in accordance with the PRISMA 2020 guidelines (24) and recommendations from the Cochrane Handbook (25). The protocol was preregistered with PROSPERO (CRD420261296015).

### Database search

Eligible studies were retrieved through an extensive literature search of PubMed, Embase, and Web of Science using a broad set of predefined search terms, which included: (1) “prediabetes” OR “pre-diabetes” OR “prediabetic” OR “pre-diabetic” OR “prediabetic state” OR “borderline diabetes” OR “impaired fasting glucose” OR “impaired glucose tolerance” OR “IFG” OR “IGT” OR “fasting glucose” OR “HbA1c”; and (2) “sleep disordered breathing” OR “sleep breathing disorders” OR “sleep apnea syndrome” OR “obstructive sleep apnea” OR “obstructive sleep apnea syndrome” OR “obstructive sleep hypopnea syndrome” OR “OSAHS” OR “OSAS” OR “sleep apnea”. The search was restricted to human research and full-text articles published in English in peer-reviewed journals. To supplement the electronic search, reference lists of pertinent original studies and reviews were manually examined to identify additional eligible publications. All databases were searched from their inception through December 28, 2025. Detailed search strategies for each database are provided in **Supplementary File 1**.

### Study inclusion and exclusion

Study eligibility was defined according to the PICOS framework:

P (Population): Adult participants (≥18 years) from community-based or clinical populations with clearly defined glycemic status, including individuals with prediabetes and normoglycemia.

I (Exposure): Prediabetes, defined according to recognized diagnostic criteria, including IFG, IGT, mildly elevated HbA1c, or composite definitions consistent with international guidelines.

Comparator (C): Individuals with normoglycemia.

Outcomes (O): Presence or prevalence of OSAS, diagnosed using objective measures such as polysomnography or portable sleep monitoring via apnea–hypopnea index (AHI)–based definitions.

S (Study design): Observational studies, including cross-sectional studies, case-control studies, and cohort studies.

Studies were excluded if they met any of the following criteria: (1) reviews, meta-analyses, editorials, letters, conference abstracts, case reports, case series, or animal studies; (2) studies conducted exclusively in pediatric or adolescent populations; (3) studies without a clear or standardized definition of prediabetes or objectively diagnosed OSAS; (4) studies in which prediabetes and diabetes were analyzed as a combined exposure without separate data for prediabetes; (5) studies lacking an appropriate normoglycemic comparison group; (6) studies that did not report effect estimates or provide sufficient data to calculate the association between prediabetes and OSAS; and (7) duplicate publications derived from the same population, in which case the study with the largest sample size was included.

### Study quality evaluation

Two reviewers independently performed the literature search, study screening, quality appraisal, and data extraction, with any disagreements resolved through discussion and consensus with the corresponding author. Study quality was assessed using the Newcastle–Ottawa Scale (NOS) (26), which evaluates selection, adjustment for confounding, and outcome assessment, yielding scores from 1 to 9, with higher scores indicating better methodological rigor. Studies scoring ≥ 8 were classified as high quality.

### Data collection

Extracted data included study-level information (author, publication year, country, and design), participant characteristics (source of the population, sample size, mean age, sex distribution, and mean body mass index [BMI] at baseline), exposure characteristics (diagnostic criteria for prediabetes and number of participants with prediabetes), outcome characteristics (diagnostic methods for OSAS and number of participants with OSAS), and variables adjusted when the association between prediabetes and OSAS was analyzed.

### Statistical analysis

The association between prediabetes and OSAS was summarized as odds ratios (ORs) with corresponding 95% confidence intervals (CIs), comparing participants with prediabetes and those with normoglycemia. When necessary, ORs and standard errors were derived from reported CIs or p-values and subsequently log-transformed to stabilize variance and approximate normality (25). Between-study heterogeneity was examined using the Cochrane Q statistic and the I² metric (27), with thresholds of < 25%, 25–75%, and > 75% interpreted as low, moderate, and high heterogeneity, respectively. Because the included studies differed in population characteristics, recruitment settings, and diagnostic criteria for prediabetes, a random-effects model was applied to account for potential between-study heterogeneity and to provide more conservative pooled estimates (25). Robustness of the overall effect was evaluated through leave-one-out sensitivity analyses (28). Prespecified subgroup analyses were performed to explore potential sources of heterogeneity and to evaluate whether study characteristics influenced the observed associations. These subgroup analyses were selected based on epidemiological and clinical considerations, including geographic region (Asian vs. Western), population source (community-derived population or those at high risk for OSAS), age distribution, sex composition, mean BMI, diagnostic criteria for prediabetes (single criteria vs. combined criteria), diagnostic methods for OSAS (polysomnography vs. portable sleep monitor), and adjustment for BMI (yes vs. no). For continuous moderators, subgroups were formed using median values as cutoff points to balance the numbers of study available in each subgroup. Potential publication bias was evaluated using funnel plot symmetry, visual inspection, and Egger’s regression test (29). A two-sided *p*-value < 0.05 was considered statistically significant. All analyses were performed using RevMan (version 5.3; Cochrane Collaboration, Oxford, UK) and Stata (version 17.0; StataCorp, College Station, TX, USA).

## Results

### Study inclusion

**Figure 1:**
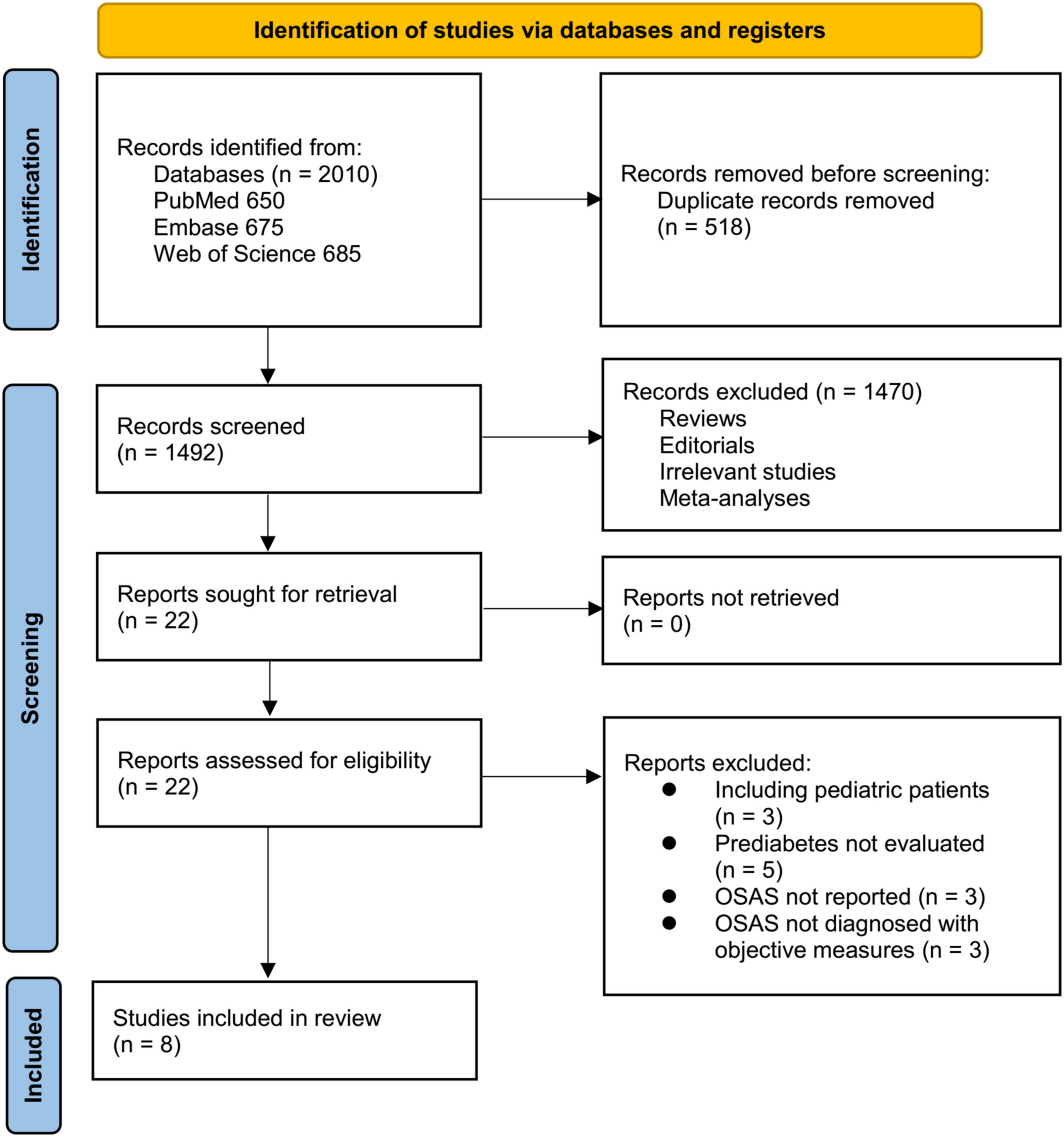
Flowchart of database search and study inclusion.

**Figure 1** depicts the study selection workflow. A total of 2010 records were retrieved from the three databases, of which 518 duplicates were removed. Screening of titles and abstracts resulted in the exclusion of 1470 records that did not meet the eligibility criteria. The full texts of the remaining 22 articles were evaluated independently by two reviewers, and 14 were excluded for reasons shown in **Figure 1**. Ultimately, eight studies met all criteria and were included in the quantitative synthesis (14–21).

### Overview of study characteristics

The characteristics of the included studies are summarized in **Table 1**. A total of eight observational studies (14–21), all adopting cross-sectional designs, were included in this meta-analysis. These studies were conducted across multiple geographic regions, including Europe, East Asia, and North America, and were derived from diverse and well-defined data sources. Several studies were based on large community-based epidemiological cohorts or population studies (16, 18, 19, 21), such as the Korean Genome and Epidemiology Study and the Hispanic Community Health Study/Study of Latinos, while others were conducted in clinical populations referred for sleep evaluation (14, 15, 17, 20). Overall, 18,040 participants were included, with sample sizes ranging from 137 to 13,394 participants. The inclusion of both population-based cohorts and clinical cohorts enhances the representativeness of the evidence base while allowing evaluation of the association across different population settings. The mean age of participants ranged from 27.8 to 67.0 years, and the proportion of men varied between 26.0% and 80.0%. Mean BMI ranged from 24.8 to 46.9 kg/m², although BMI data were not reported in one large population-based study (21). Study populations were heterogeneous and included community-based adults (16, 18, 19, 21), extremely obese individuals (14), overweight or obese young adults (17), and patients clinically suspected of OSAS referred for polysomnography (15, 20).

**Table 1:** Characteristics of the included studies.

Study	Design	Country	Participant characteristics	Sample size	Mean age (years)	Men (%)	Mean BMI (kg/m^2^)	Diagnostic criteria for PreD	No. of participants with PreD	Diagnostic criteria for OSAS	No. of participants with OSAS	Variables matched or adjusted
Fredheim 2011 (14)	CS	Norway	Extremely obese population	137	43.0	26.0	46.9	IFG and/or IGT	58	Portable sleep monitor, AHI ≥ 5 events/hour	84	Age, sex, BMI, HOMA-IR, and hsCRP
Tamura 2012 (15)	CS	Japan	Patients clinically suspected of OSAS referring for PSG	330	60.0	80.0	24.8	IGT	111	PSG, AHI ≥ 5 events/hour	292	Age and sex
Kim 2013 (16)	CS	South Korea	Community-based adults (≥40 years) from the Korean Genome and Epidemiology Study	1344	57.7	52.5	24.8	IFG and/or IGT	424	Portable sleep monitor, AHI ≥ 5 events/hour	625	Age, sex, alcohol, smoking, exercise, hypertension/CVD, dyslipidemia medication, and BMI
Li 2020 (17)	CS	China	Overweight/obese (BMI ≥ 25 kg/m²) nondiabetic young adults (18–45 years) from a sleep disorders center	422	27.8	61.9	34.8	IFG and/or IGT	147	PSG, AHI ≥ 5 events/hour	257	Age and sex
Sanchez 2022 (18)	CS	Spain	Community-based middle-aged adults (men 45–65, women 50–70	966	58.0	48.7	28.5	HbA1c 5.7–6.4%	311	PSG, AHI ≥ 5 events/hour	701	Age, sex, and BMI
Chen 2023 (19)	CS	USA	Community based adults aged 45–84 years	1301	67.0	48.9	27.3	IFG	330	PSG, AHI ≥ 5 events/hour	887	Age, sex, race, drinking, smoking, physical activity, eGFR, lipids, and CVD history
López-Cepero 2024 (21)	CS	USA	Community based hispanic/Latino adults aged 18–74 years	13394	41.2	47.3	NR	IFG and/or IGT	4835	Portable sleep monitor, AHI ≥ 5 events/hour	4075	Age, sex, heritage, education, smoking, hs-CRP, BMI, sleep duration, and hypertension
Abelleira 2024 (20)	CS	Spain	Patients clinically suspected of OSAS referring for PSG	146	47.7	61.3	30.2	IFG and/or HbA1c 5.7–6.4%	109	PSG, AHI ≥ 5 events/hour	208	Age, sex, and BMI

AHI, apnea–hypopnea index; BMI, body mass index; CS, cross-sectional; CVD, cardiovascular disease; eGFR, estimated glomerular filtration rate; HbA1c, glycated hemoglobin; HOMA-IR, homeostasis model assessment of insulin resistance; hsCRP, high-sensitivity C-reactive protein; IFG, impaired fasting glucose; IGT, impaired glucose tolerance; NR, not reported; OSAHS, obstructive sleep apnea–hypopnea syndrome; OSAS, obstructive sleep apnea syndrome; PreD, prediabetes; PSG, polysomnography.

Prediabetes was defined using established diagnostic criteria, including IFG (19), IGT (15), HbA1c of 5.7–6.4% (18), or combinations of these definitions (14, 16, 17, 20, 21) The number of participants with prediabetes ranged from 58 to 4,835 across studies, and a total of 6,325 (35.1%) participants were with prediabetes in total. OSAS was diagnosed using either attended polysomnography (15, 17–20) or validated portable sleep monitoring devices (14, 16, 21), with a consistent diagnostic threshold of AHI ≥ 5 events per hour applied across all studies. Accordingly, 7,129 (39.5%) subjects were diagnosed as OSAS. All studies performed multivariable adjustment or matching for potential confounders, at least for age and sex, and some studies (14, 16, 18–21) also adjusted BMI, lifestyle factors, inflammatory markers, cardiometabolic comorbidities, or renal function indicators, to a varying degree.

### Study quality evaluation

Study quality was assessed using the NOS, and the results are presented in **Table 2**. Total NOS scores ranged from 7 to 9, indicating overall moderate to high methodological quality among the included studies. Four studies (14, 16, 19, 21) achieved the maximum score of 9, reflecting adequate case definition, good representativeness, appropriate control selection, reliable ascertainment of OSAS exposure, consistent outcome assessment methods, and comprehensive adjustment for major confounding variables. Two studies received NOS scores of 8 (18, 20), primarily due to incomplete reporting of non-response rates, despite otherwise strong methodological characteristics. Another two studies obtained a score of 7, mainly because of limited adjustment for additional confounders beyond age and sex, and incomplete reporting of non-response rates (15, 17). Importantly, no study was considered to be of low quality, and all included studies met fundamental criteria for appropriate exposure definition, valid outcome assessment, and comparability between prediabetes and normoglycemic groups.

**Table 2:** Study quality evaluation via the Newcastle-Ottawa scale.

Study	Adequate definition of cases	Representativeness of cases	Selection of controls	Definition of controls	Control for age and sex	Control for other confounders	Exposure ascertainment	Same methods for events ascertainment	Non-response rates	Total
Fredheim 2011 (14)	1	1	1	1	1	1	1	1	1	9
Tamura 2012 (15)	1	1	1	1	1	0	1	1	0	7
Kim 2013 (16)	1	1	1	1	1	1	1	1	1	9
Li 2020 (17)	1	1	1	1	1	0	1	1	0	7
Sanchez 2022 (18)	1	1	1	1	1	1	1	1	0	8
Chen 2023 (19)	1	1	1	1	1	1	1	1	1	9
López-Cepero 2024 (21)	1	1	1	1	1	1	1	1	1	9
Abelleira 2024 (20)	1	1	1	1	1	1	1	1	0	8

### Association between prediabetes and OSAS

**Figure 2:**
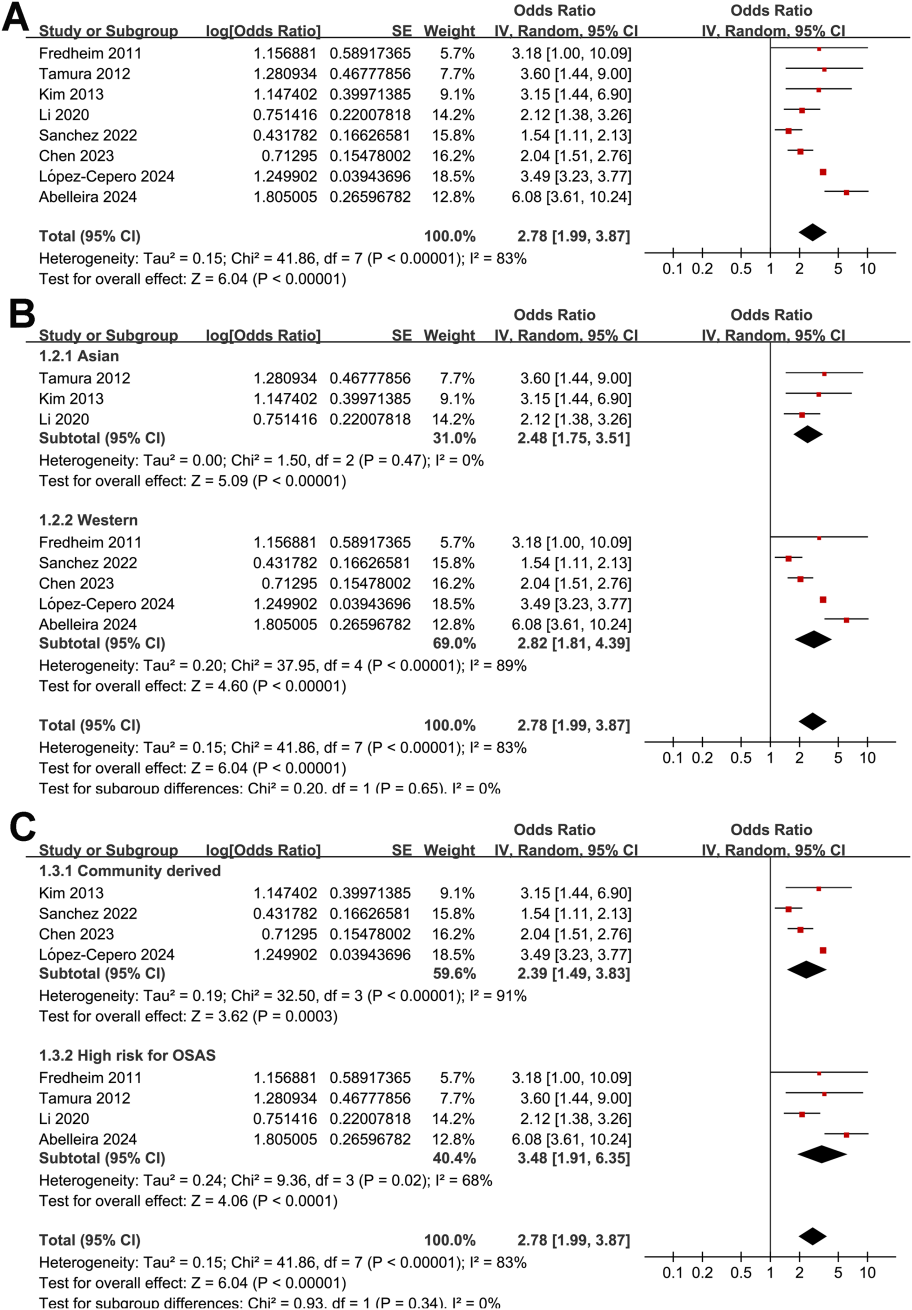
Forest plots for the meta-analysis of the association between prediabetes and OSAS in adult population. **(A)** forest plots of the overall meta-analysis; **(B)** forest plots of the subgroup analysis according to study country; and **(C)** forest plots of the subgroup analysis according to the source of the population.

**Figure 3:**
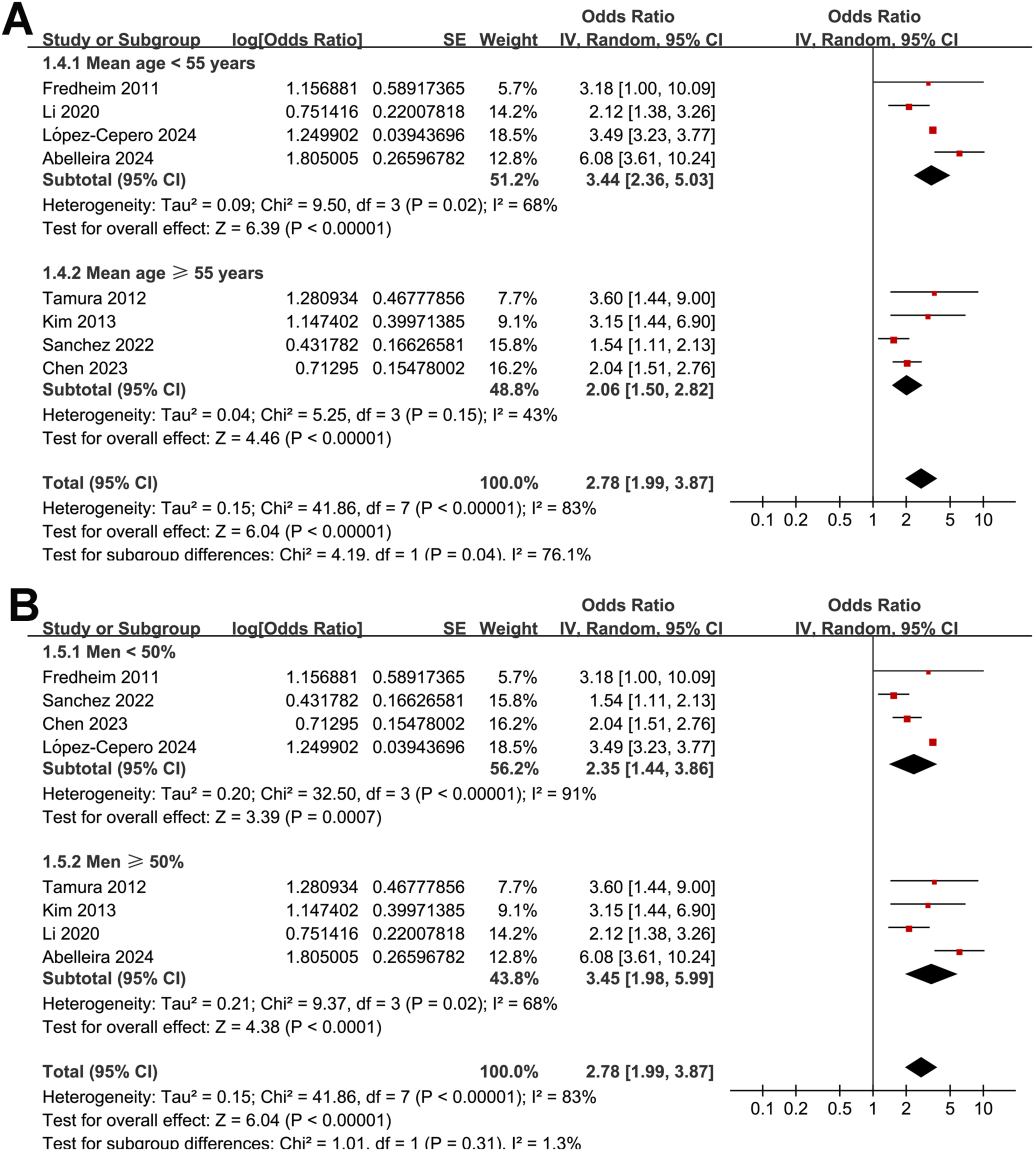
Forest plots for the subgroup analysis of the association between prediabetes and OSAS in adult population. **(A)** subgroup analysis according to the mean ages of the participants; and **(B)** subgroup analysis according to the proportions of men.

**Figure 4:**
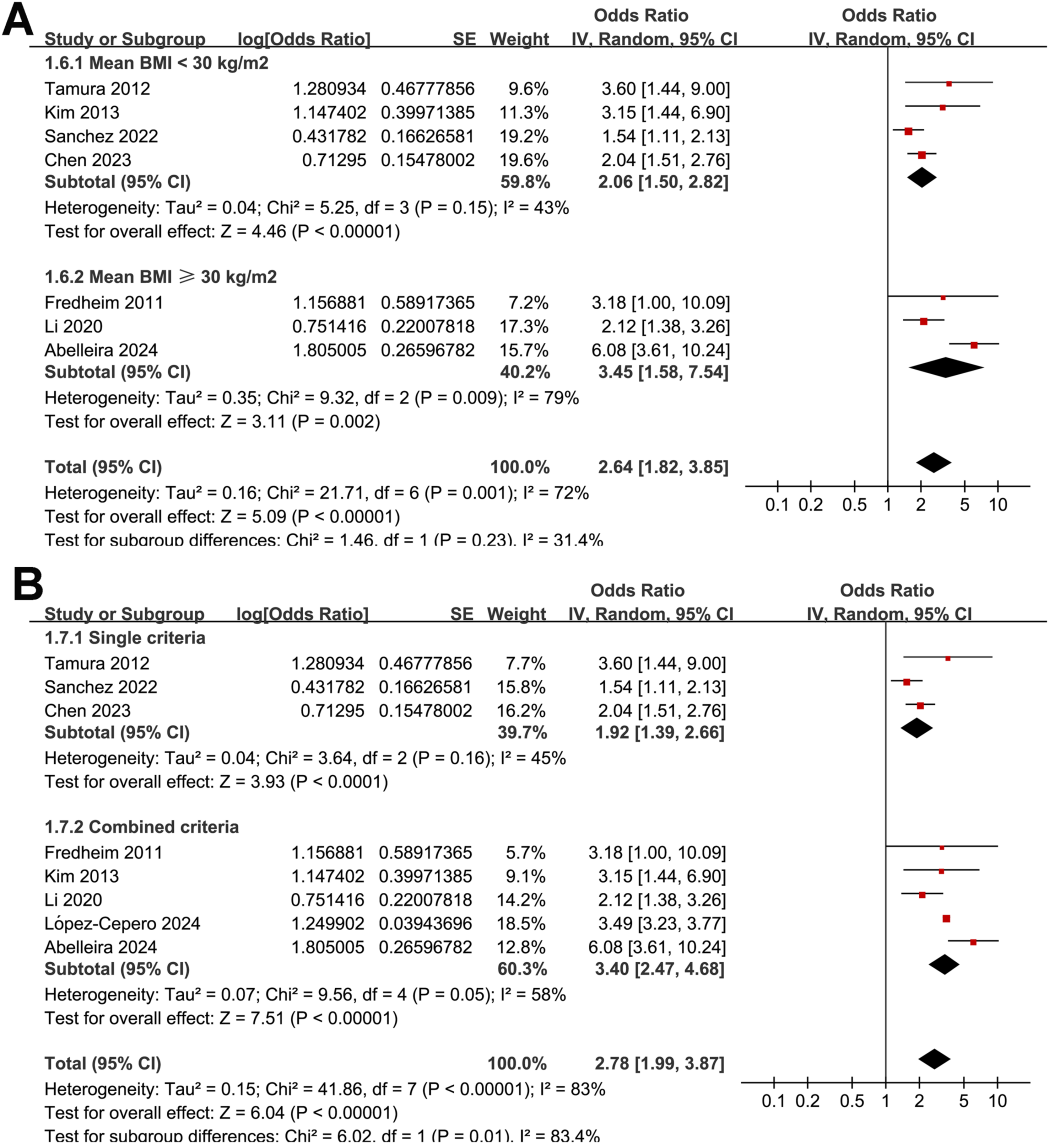
Forest plots for the subgroup analysis of the association between prediabetes and OSAS in adult population. **(A)** subgroup analysis according to the mean BMI of the participants; and **(B)** subgroup analysis according to the diagnostic criteria for prediabetes.

**Figure 5:**
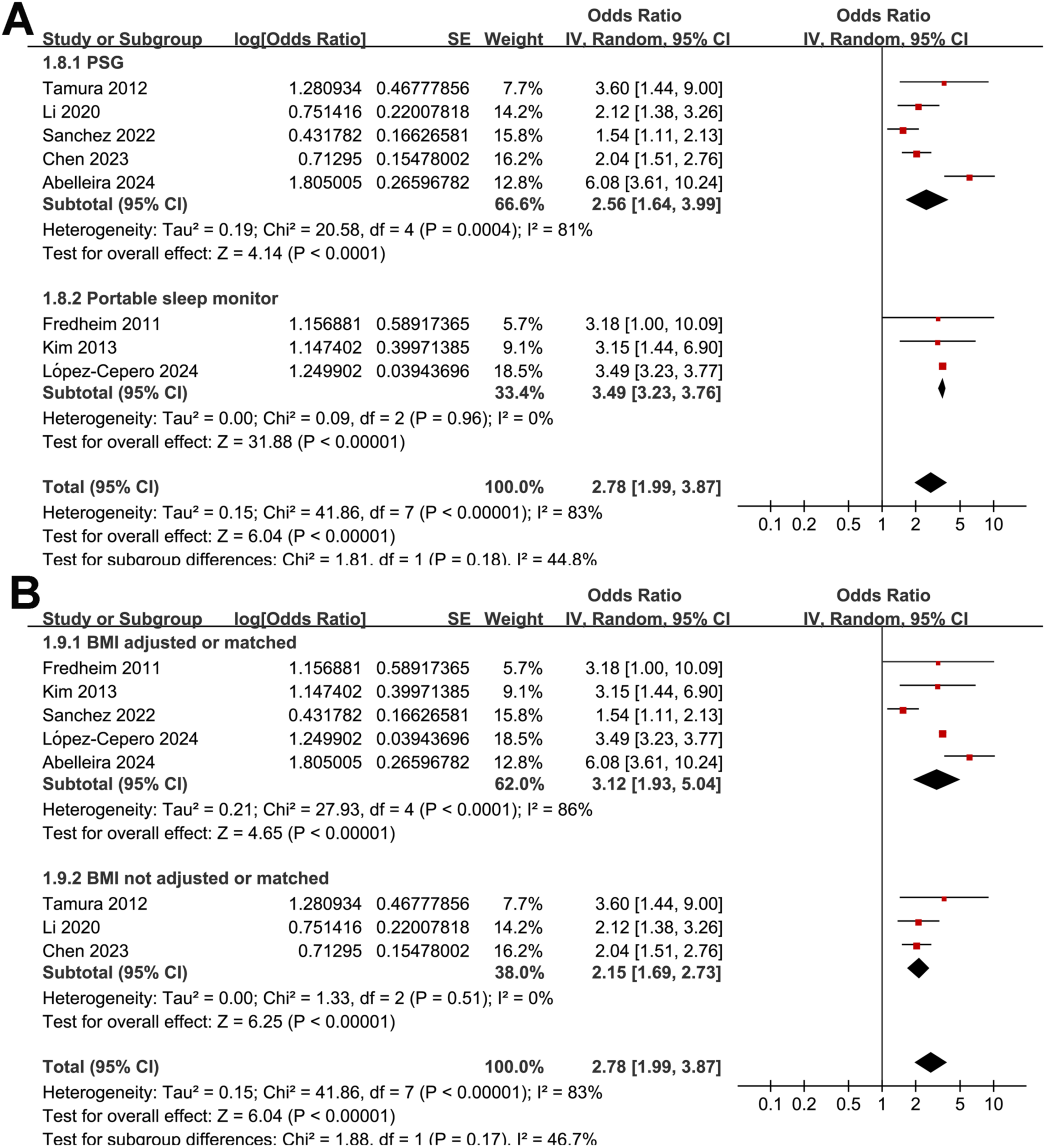
Forest plots for the subgroup analysis of the association between prediabetes and OSAS in adult population. **(A)** subgroup analysis according to the diagnostic methods for OSAS; and **(B)** subgroup analysis according to the adjustment of BMI;.

Pooled results of the eight studies (14–21) showed that compared to subjects with normoglycemia, those with prediabetes were associated with higher prevalence of OSAS (OR: 2.78, 95% CI: 1.99 to 3.87, *p* < 0.001; **Figure 2A**) with substantial heterogeneity (*p* for Cochrane Q test < 0.001; I^2^ = 83%). Sensitivity analysis by excluding one study at a time showed consistent results (OR: 2.47 to 3.08, *p* all < 0.05). In particular, sensitivity analysis limited to high–quality studies with NOS of 8 or 9 showed similar results (OR: 3.12, 95% CI: 1.93 to 5.04, *p* < 0.001; I^2^ = 86%). Further subgroup analyses demonstrated consistent associations between prediabetes and OSAS in studies from Asian and Western countries (OR: 2.48 vs. 2.82, *p* for subgroup difference = 0.65; **Figure 2B**), and in community-derived population and people at high risk for OSAS (OR: 2.39 vs. 3.48, *p* for subgroup difference = 0.34; **Figure 2C**). Interestingly, a stronger association between prediabetes and OSAS was observed in participants with mean age < 55 years as compared to those ≥ 55 years (OR: 3.44 vs. 2.06, *p* for subgroup difference = 0.04; **Figure 3A**). Similar results were observed between studies with the proportion of men < 50% and ≥ 50% (OR: 2.35 vs. 3.45, *p* for subgroup difference = 0.31; **Figure 3B**), and between studies with the mean BMI of the population < 30 kg/m^2^ and ≥ 30 kg/m^2^ (OR: 2.06 vs. 3.45, *p* for subgroup difference = 0.23; **Figure 4A**). A stronger association between prediabetes and OSAS was observed for studies with prediabetes diagnosed with composite criteria as compared to those based on single criteria (OR: 3.40 vs. 1.92, *p* for subgroup difference = 0.01; **Figure 4B**). Finally, the results seem to be similar between studies with OSAS diagnosed with polysomnography or portable sleep monitoring (OR: 2.56 vs. 3.49, *p* for subgroup difference = 0.18; **Figure 5A**), and between studies with or without the adjustment of BMI (OR: 3.12 vs. 2.15, *p* for subgroup difference = 0.17; **Figure 5B**).

### Publication bias

**Figure 6:**
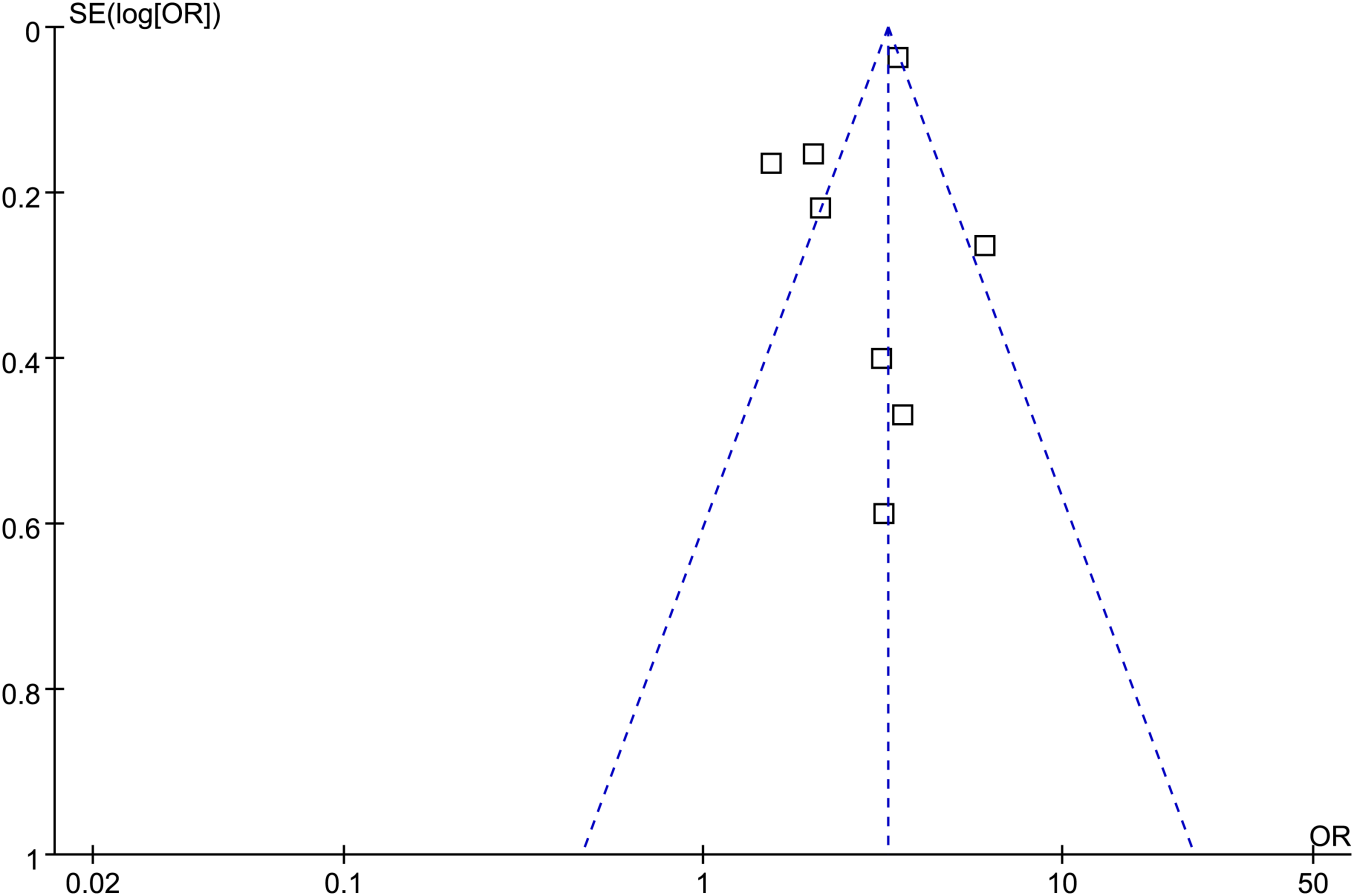
Funnel plots estimating the potential publication bias underlying the meta-analysis of the association between prediabetes and OSAS in adult population.

**Figure 6** displays the funnel plots evaluating potential publication bias for the meta-analyses of the association between prediabetes and OSAS in adults. No obvious asymmetry was observed on inspection. Egger’s regression test also did not show a significant risk of publication bias (*p* = 0.29). However, given the limited number of included studies (k = 8), the publication bias could not be excluded completely.

## Discussion

The present meta-analysis provides a comprehensive synthesis of current evidence regarding the association between prediabetes and OSAS in adult populations. By integrating data from diverse geographic regions and study settings, our findings consistently indicate that prediabetes is closely associated with a higher prevalence of OSAS. Importantly, this association remained robust across multiple sensitivity analyses and prespecified subgroup analyses, supporting the stability of the observed relationship. Rather than suggesting causality, these results highlight the frequent coexistence of early glucose dysregulation and sleep-disordered breathing and emphasize their potential interaction within a shared metabolic milieu.

Several biological mechanisms may underlie the observed association between prediabetes and OSAS. Individuals with prediabetes commonly exhibit insulin resistance, low-grade systemic inflammation, and autonomic imbalance, all of which may contribute to upper airway vulnerability and impaired ventilatory control during sleep (30, 31). Hyperinsulinemia and altered glucose metabolism can promote sympathetic nervous system activation and fluid redistribution to the neck during the nocturnal period, increasing upper airway collapsibility (32, 33). In addition, inflammatory mediators such as interleukin-6 and tumor necrosis factor-α, which are elevated in prediabetes, may adversely affect neuromuscular control of the upper airway (34). Although these mechanisms remain incompletely elucidated, they provide biological plausibility for the frequent coexistence of prediabetes and OSAS observed across epidemiological studies. Beyond potential direct links, prediabetes and OSAS also share multiple common pathogenic pathways and risk factors. Obesity, particularly visceral adiposity, represents a central contributor to both conditions through its effects on insulin resistance, systemic inflammation, and upper airway anatomy (35). Additional shared factors include aging, sedentary lifestyle, dyslipidemia, and cardiometabolic comorbidities (36, 37). These overlapping risk profiles likely amplify the clustering of prediabetes and OSAS within the same individuals. However, the persistence of a significant association in studies that adjusted or matched for BMI suggests that the relationship cannot be explained by obesity alone, supporting a more complex metabolic interaction beyond simple confounding.

The subgroup analyses offer further insights into the nature of this association. Notably, the relationship between prediabetes and OSAS was observed across both community-derived populations and populations at high risk for sleep apnea, indicating that the association is not restricted to sleep clinic–based cohorts. The influence of BMI was particularly informative: although stronger associations seemed numerically observed in studies involving more obese populations, the association remained significant regardless of mean BMI category and persisted in studies that adjusted for BMI. These findings suggest that while adiposity may strengthen the relationship, prediabetes is associated with OSAS even after accounting for body weight, underscoring the potential metabolic contribution independent of obesity. Age appeared to modify the strength of the association, with a more pronounced relationship observed in studies involving younger populations. One possible explanation is that younger individuals with prediabetes may exhibit a more prominent metabolic phenotype characterized by insulin resistance and sympathetic overactivity, which could predispose them to early sleep-disordered breathing. In contrast, in older populations, the contribution of age-related structural airway changes and comorbidities may dilute the relative impact of glycemic status on OSAS prevalence. Another possible explanation is that the clinical significance of AHI values may differ across age groups. In older adults, mild elevations in AHI are relatively common and may reflect age-related physiological changes rather than clinically significant sleep-disordered breathing (38). Consequently, higher AHI thresholds may be required in older populations to demonstrate meaningful associations with cardiovascular and metabolic risk (38). This phenomenon may partly attenuate the observed association between prediabetes and OSAS in studies involving older participants. These findings highlight the importance of considering age-related differences when interpreting AHI-based definitions of sleep apnea and their metabolic implications. In addition, this age-related difference also highlights the importance of early metabolic disturbances and suggests that prediabetes at a younger age may identify a subgroup particularly vulnerable to sleep-related breathing abnormalities. The diagnostic criteria used to define prediabetes also influenced the magnitude of the association. Studies employing composite definitions incorporating fasting glucose, glucose tolerance testing, or HbA1c demonstrated stronger associations than those relying on a single criterion. This finding likely reflects more accurate classification of dysglycemia and better capture of individuals with sustained metabolic impairment. Composite diagnostic approaches may identify a metabolically more advanced prediabetic state, in which insulin resistance, inflammation, and neurohormonal dysregulation are more pronounced, thereby strengthening the observed relationship with OSAS (39).

Several strengths of this meta-analysis should be acknowledged. First, the analysis incorporated data from multiple well-characterized populations across different regions, including large community-based epidemiological cohorts and clinical sleep cohorts, thereby enhancing the representativeness and generalizability of the evidence base. Second, an up-to-date and comprehensive literature search was conducted across major databases, ensuring broad coverage of relevant studies. Third, only studies using objective diagnostic methods for OSAS were included, enhancing the reliability of outcome assessment. Fourth, extensive sensitivity and subgroup analyses were performed, which consistently supported the robustness of the main findings. Together, these methodological strengths increase confidence in the observed association and reduce the likelihood that the results are driven by a single study or analytic approach. Nevertheless, several important limitations warrant careful consideration. All included studies were cross-sectional in design, precluding assessment of temporal relationships or causal inference between prediabetes and OSAS. As a result, the directionality of the association cannot be determined, and reverse causation cannot be excluded. Substantial heterogeneity was observed across studies, likely reflecting differences in population characteristics, recruitment settings, and diagnostic criteria for prediabetes. Although subgroup analyses explored several of these factors, residual heterogeneity could not be fully explained, particularly in the absence of individual participant data. In addition, unmeasured or inconsistently adjusted confounders, such as visceral adiposity, sleep duration, medication use, and lifestyle behaviors, may have influenced the observed associations. Another limitation is that the association between prediabetes and different severity levels of OSAS could not be evaluated. Most included studies reported OSAS based on the conventional diagnostic threshold of an AHI ≥ 5 events/hour, and effect estimates stratified by severity categories (e.g., AHI ≥ 15 events/hour) were not consistently available. Therefore, the potential influence of moderate-to-severe OSAS on the strength of the association could not be examined and warrants further investigation in future studies with standardized severity reporting. Finally, because the meta-analysis relied on aggregate data, detailed mechanistic pathways and dose–response relationships could not be evaluated.

From a clinical perspective, the findings of this study should be interpreted cautiously and viewed as evidence of association rather than causation. Nonetheless, the consistent coexistence of prediabetes and OSAS has important clinical implications. Prediabetes represents a critical window for metabolic intervention, during which progression to overt diabetes may be preventable (40). Given the established adverse effects of OSAS on insulin resistance and glycemic control, unrecognized sleep apnea in individuals with prediabetes may accelerate metabolic deterioration (41). Therefore, heightened awareness and consideration of OSAS screening in prediabetic populations may facilitate earlier identification of high-risk individuals and support integrated metabolic and sleep management strategies. Future research should focus on longitudinal cohort studies to clarify temporal relationships between prediabetes and OSAS and to determine whether one condition predisposes to the development or progression of the other. Interventional studies evaluating the metabolic effects of OSAS treatment in individuals with prediabetes are also needed to establish whether early management of sleep-disordered breathing can modify glycemic trajectories. Such evidence would be critical for informing preventive strategies and clinical guidelines.

## Conclusions

In conclusion, this meta-analysis demonstrates that prediabetes is significantly associated with a higher prevalence of OSAS in adults. Although causality cannot be inferred, the consistent association observed across diverse populations highlights the close interaction between early glucose dysregulation and sleep-disordered breathing. These findings underscore the importance of recognizing OSAS in individuals with prediabetes and provide a rationale for integrated screening approaches aimed at preventing further deterioration of metabolic health.

## Data Availability

The original contributions presented in the study are included in the article/**Supplementary Material**. Further inquiries can be directed to the corresponding author.
