# Supplementary material for: The association between prediabetes and obstructive sleep apnea syndrome: a meta-analysis
Source: Front Endocrinol (Lausanne). 2026 Jul 30;17:1798827. doi: 10.3389/fendo.2026.1798827 (PMC13467845; doi:10.3389/fendo.2026.1798827)
Supplement: Supplementary file 1 [file DataSheet1.docx]

**Detailed search strategy for each database**

**PubMed**

("Prediabetic State"[Mesh] OR prediabetes[Title/Abstract] OR pre-diabetes[Title/Abstract] OR prediabetic[Title/Abstract] OR pre-diabetic[Title/Abstract] OR "prediabetic state"[Title/Abstract] OR "borderline diabetes"[Title/Abstract] OR "impaired fasting glucose"[Title/Abstract] OR "impaired glucose tolerance"[Title/Abstract] OR IFG[Title/Abstract] OR IGT[Title/Abstract] OR "fasting glucose"[Title/Abstract] OR HbA1c[Title/Abstract]) AND ("Sleep Apnea Syndromes"[Mesh] OR "Sleep Apnea, Obstructive"[Mesh] OR "sleep disordered breathing"[Title/Abstract] OR "sleep breathing disorders"[Title/Abstract] OR "sleep apnea syndrome"[Title/Abstract] OR "obstructive sleep apnea"[Title/Abstract] OR "obstructive sleep apnea syndrome"[Title/Abstract] OR "obstructive sleep hypopnea syndrome"[Title/Abstract] OR OSAHS[Title/Abstract] OR OSAS[Title/Abstract] OR "sleep apnea"[Title/Abstract])

**Embase**

('prediabetes'/exp OR prediabetes:ti,ab OR 'pre diabetes':ti,ab OR prediabetic:ti,ab OR 'prediabetic state':ti,ab OR 'borderline diabetes':ti,ab OR 'impaired fasting glucose':ti,ab OR 'impaired glucose tolerance':ti,ab OR IFG:ti,ab OR IGT:ti,ab OR 'fasting glucose':ti,ab OR HbA1c:ti,ab) AND ('sleep disordered breathing'/exp OR 'obstructive sleep apnea'/exp OR 'sleep apnea syndrome':ti,ab OR 'obstructive sleep apnea':ti,ab OR 'obstructive sleep apnea syndrome':ti,ab OR 'obstructive sleep hypopnea syndrome':ti,ab OR OSAHS:ti,ab OR OSAS:ti,ab OR 'sleep apnea':ti,ab)

**Web of Science**

TS = (prediabetes OR "pre-diabetes" OR prediabetic OR "pre-diabetic" OR "prediabetic state" OR "borderline diabetes" OR "impaired fasting glucose" OR "impaired glucose tolerance" OR IFG OR IGT OR "fasting glucose" OR HbA1c) AND TS = ("sleep disordered breathing" OR "sleep breathing disorders" OR "sleep apnea syndrome" OR "obstructive sleep apnea" OR "obstructive sleep apnea syndrome" OR "obstructive sleep hypopnea syndrome" OR OSAHS OR OSAS OR "sleep apnea")
